# Differentiation of Tuberculosis Strains in a Population with Mainly Beijing-family Strains

**DOI:** 10.3201/eid1209.041263

**Published:** 2006-09

**Authors:** Vladyslav Nikolayevskyy, Krishna Gopaul, Yanina Balabanova, Timothy Brown, Ivan Fedorin, Francis Drobniewski

**Affiliations:** * Barts and the London School of Medicine, University of London, London, United Kingdom;; † Samara Regional Tuberculosis Service, Samara, Russia

**Keywords:** *Mycobacterium tuberculosis*, Beijing family, genotyping, Mycobacterial interspersed repetitive units

## Abstract

A high prevalence of tuberculosis (TB) isolates that are genetically homogenous and from the Beijing family has been reported in Russia. To map TB transmission caused by these strains, new genotyping systems are needed. Mycobacterial interspersed repetitive units (MIRUs) offer the possibility of rapid PCR-based typing with comparable discrimination to IS*6110* restriction fragment length polymorphism techniques. Spoligotyping and detection of IS*6110* insertion in the *dnaA-dnaN* region were used to identify Beijing strains in 187 *Mycobacterium tuberculosis* isolates from Samara, Russia. The Beijing isolates were analyzed by using 12-MIRU and 3–exact tandem repeats (ETR) loci and by an expanded set of 10 additional variable number tandem repeats loci. The expanded set of 25 MIRUs provided better discrimination than the original set of 15 (Hunter-Gaston diversity index 0.870 vs 0.625). Loci MIRU 26, 1982, and 3232 were the most polymorphic in Beijing isolates.

## Abstract 2

A new panel of 25 VNTR-MIRU loci differentiates Beijing-family TB strains better than a panel of 15.

Rising rates of tuberculosis (TB) (*1*) and increasing resistance of drugs are substantial barriers to successful patient management and TB control programs. Russia, named by the World Health Organization as one of the 22 countries with the highest TB prevalence, has seen increasing rates of TB and HIV during the past decade (*2**–**6*) and high levels of resistance, particularly to multiple drugs (*7**,**8*).

The high discriminatory power of restriction fragment length polymorphism (RFLP) analysis based on the insertion sequence IS*6110* has provided the backbone of these analyses (*9*). Unfortunately, the technique is time-consuming, technically demanding, and insufficiently discriminating when used alone with isolates containing <5 IS*6110* sequences in the genome (*10**,**11*). Isolates with low numbers of copies account for as much as 20% of TB isolates in some populations (*12*).

Techniques based on PCR amplification of repetitive sequences are more rapid than RFLP, but their discriminatory power is usually lower. Among these techniques, spoligotyping is widely used to differentiate strains belonging to the *Mycobacterium tuberculosis* complex. Spoligotyping has been particularly useful for identifying strains belonging to the Beijing/W family of *M. tuberculosis* because of the characteristic spoligotyping pattern with the absence of spacers 1–34 in the direct repeat (DR) region of the *M. tuberculosis* genome (*13*). Beijing family strains are dominant across many Asian and former Soviet Union countries, and W strains are responsible for outbreaks of multidrug-resistant TB in the United States. These strains are now considered to be members of the same phylogenetic lineage, sharing key characteristics such as a similar RFLP pattern of 15–26 bands, IS*6110* insertions in the *dnaA*-*dnaN* and NTF-1 chromosomal regions, a characteristic pattern of single nucleotide polymorphism, and a spoligotyping pattern with the presence of spacers 35–43 and absence of spacers 1–34 in the DR region of the *M. tuberculosis* genome (*14**–**17*). Detecting the IS*6110* insertion in the *dnaA*-*dnaN* intergenic region may also identify the Beijing/W genotype (*14**,**15*).

Several studies have shown that a high proportion of TB isolates in Russia (particularly those that are drug resistant) belong to the Beijing family (*7**,**8**,**18*). To assess TB transmission in Russia, any genotyping system must be able to discriminate among Beijing strains. The identification of variable number tandem repeats (VNTRs) (*19*) in *M. tuberculosis* has offered the possibility of rapid amplification-based techniques with comparable discrimination to RFLP-IS*6110* typing (*20**–**23*).

As with other PCR-based genotyping techniques, VNTR analysis uses small quantities of crude bacterial lysates; it is less labor-intensive than RFLP-IS*6110* typing, and automation is relatively straightforward (*22*). Moreover, determination of a limited number of polymorphic loci can provide sufficient discriminative power for a given local population and may increase the cost-effectiveness of molecular typing. Several panels of *M. tuberculosis* VNTRs have been used with some success previously: exact tandem repeats (ETRs) (*19*), MIRUs (*21**,**22*), and 2 panels of loci known as QUB and Mtub (*24**–**27*). Our aim was to determine the discriminative power of an expanded set of 25 VNTR loci when applied to TB strains in Russia where Beijing strains dominate.

## Methods

### *M. tuberculosis* Strains

A total of 187 *M. tuberculosis* strains were analyzed. They were selected from 880 *M. tuberculosis* strains isolated from patients (1 isolate per patient) with radiologically confirmed pulmonary TB, identified from all TB treatment facilities across Samara Oblast in central Russia (12 civilian TB hospitals and dispensaries and 1 prison TB hospital) during 2001–2002. The test panel included 138 (33.7%) of 409 strains isolated from patients in the civilian TB hospitals and dispensaries and 49 (10.4%) of 471 isolates from prisoners. Within sets of cultures isolated from civilians, every third isolate was selected for this study. Because isolates from prisoners were overrepresented, every 10th isolate was selected. The representation of isolates in the test panel was approximately proportional to the number of TB isolates from each clinical site. In the Samara region, the incidence of TB at the time of the study was 86.1/100,000 population.

Patient populations are described in previous publications (*18**,**28*). All patients with TB are tested for HIV as a part of routine practice in Russia. History of vaccination with *M. bovis* BCG was confirmed by presence of an appropriate scar.

### Molecular Epidemiologic Analysis

Crude DNA extracts were obtained by heating cell suspensions with chloroform at 80°C as previously described (*29*). Spoligotyping used a standardized method (*30*) to identify isolates belonging to the Beijing family; results were confirmed by analysis of the *dnaA* to *dnaN* region in all isolates. The IS*6110* insertion in the origin of replication was detected between *dnaA* and *dnaN* genes as described previously (*15**,**16*). Briefly, PCR was performed in a 20-μL volume of 2 μL 10× PCR buffer (Bioline Ltd, London, UK); 0.5 units Taq polymerase (Bioline Ltd); 0.5 μL 2 mmol dNTP mixture (Bioline Ltd); 0.5 μL 20-μmol mix of forward and reverse primers, 15.5 μL water, and 1 μL of crude DNA extract. Thermal cycling was performed on a PerkinElmer 9700 thermocycler (PerkinElmer, Warrington, UK) as follows: 4 min at 94°C; 30 cycles of 30 s at 94°C, 30 s at 60°C, and 2 min at 72°C; followed by 7 min at 72°C and holding at 4°C. Amplification products were analyzed by electrophoresis on 1.5% agarose gel. Strains with insertion (i.e., Beijing family) yielded a product of ≈2 kb, and fragments of ≈550 bp (no insertion) indicated strains other than Beijing.

All 187 TB isolates were tested by using the set of 12-MIRU loci and the 3-ETR (A, B, and C) loci (*19**,**22*) (nos. 1–15, Table A1). Beijing isolates were further analyzed by using an additional panel of VNTR loci (0424, 0531, 1955, 1982, 2074, 2163a, 3232, 3239, 3336, and 3690, Table A1). Primers for loci at which predicted fragment size would exceed 1 kb were redesigned to enable analysis with the CEQ8000 equipment (Beckman Coulter, Fullerton, CA, USA).

Multiplex and simplex PCRs were performed, taking into consideration dye labeling and expected length of PCR products. Simplex PCR was used to amplify fragments in loci MIRU 20, ETR-C, MIRU 26, VNTR 424, VNTR 531, VNTR 1955, VNTR 1982, VNTR 2163a, VNTR 3232, VNTR 3239, and VNTR 3336. For other loci, multiplex PCR mixtures were prepared as follows: set 1 contained MIRU 4 and MIRU 16, set 2 contained MIRU 39 and ETR-A, set 3 contained MIRU 2 and MIRU 24, set 4 contained MIRU 31 and MIRU 40, set 5 contained MIRU 10 and MIRU 23, set 6 contained MIRU 27 and ETR-B, and set 7 contained VNTR 2074 and VNTR 3690.

For all mixtures, PCR was performed in 10-μL volumes containing 1 μL 10× PCR buffer (containing 1.5 mmol/L MgCl_2_, Bioline Ltd); 0.5 U Taq polymerase (Bioline Ltd); 0.25 μl 2-mmol dNTP mixture (Bioline Ltd); 0.5 μL 20-μmol mixture of forward and reverse primers as described above, 7.0 μL water, and 1 μL DNA extract. For loci VNTR 424, VNTR 531, VNTR 1955, VNTR 1982, VNTR 2074, VNTR 2163a, VNTR 3232, VNTR 3239, VNTR 3336, and VNTR 3690, the mixture also contained 0.5 μL dimethylsulfoxide (Sigma, Dorset, UK). Thermal cycling programs were identical for all loci, and thermal cycling was performed on a PerkinElmer 9700 thermocycler by using the following parameters: 3 min at 95°C; 30 cycles of 30 s at 95°C, 30 s at 60°C, and 60 s at 72°C; followed by 5 min at 72°C.

Automated analysis of PCR fragments length was performed by using a Beckman Coulter CEQ8000 automatic sequencer. Multiple PCR products were analyzed in capillaries as follows: capillary 1 contained PCR products for loci 2, 4, 10, 16, 23, and 24; capillary 2 for loci 27, 31, 39, ETR-A, and ETR-B; capillary 3 for loci 20, 26, and ETR-C; capillary 4 for loci 0424, 0531, and 1955; capillary 5 for loci 2974, 3232, 3239, and 3690; and capillary 6 for loci 1982, 2163a, and 3336.

Before being loaded onto the sequencer, PCR products were diluted in purified water (Sigma) as follows: products labeled with dye 2 were diluted to 10-fold; with dye 3, to 30-fold; and with dye 4, to 60-fold. A total of 1 μL of diluted PCR products mixture was added to 25 μL formamide (Beckman Coulter) containing 0.1 μL of DNA size standard 600 (Beckman Coulter), and 0.1 μL DNA size standard 640–1,000 (Bio Ventures Inc., Murfreesboro, TN, USA), labeled with dye 1. Fragment length estimation was performed by using proprietary software (Beckman Coulter).

Molecular weights of PCR-generated fragments for loci 1982 and 3232 for some isolates exceeded 1 kb, and results of automated fragment analysis were inconsistent. Molecular weights of PCR products and numbers of MIRU repeats for these strains were determined manually by electrophoresis on a 1.2% agarose gel (Agarose 1000, Invitrogen Ltd, Paisley, UK) with a 100-bp step DNA ladder as fragment size standard (Promega, Madison, WI, USA) (Figure).

**Figure:**
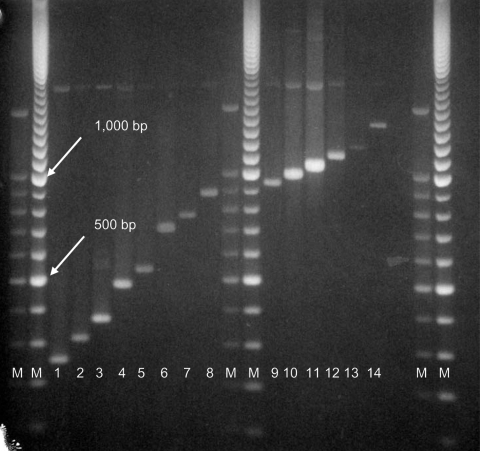
PCR analysis of VNTR3232 locus. Lane 1, 2 repeats; lane 2, 3 repeats; lane 3, 4 repeats; lane 4, 6 repeats; lane 5, 7 repeats; lane 6, 10 repeats; lane 7, 11 repeats; lane 8, 12 repeats; lane 9, 13 repeats; lane 10, 14 repeats; lane 11, 15 repeats; lane 12, 16 repeats; lane 13, 17 repeats; lane 14, 20 repeats. Lanes 1–5, strains other than Beijing; lanes 6–14, Beijing strains. M, molecular weight markers.

Automated calling (in which PCR fragment sizes and allele assignment are automatically determined) or manual determination of genotyping data (number of repeats for each loci) was entered into Microsoft Excel (Redmond, WA, USA) tables and imported for further analysis into BioNumerics software (Applied Maths, St Martens, Belgium). Genetic distance analysis and cluster comparison were done by using a categorical variable index and the unweighted pair group method with arithmetic mean algorithm. If >2 isolates possessed identical VNTR signatures, they were considered to be clustered. For discrimination analysis, Hunter-Gaston diversity index (HGDI) was calculated as described (*31*) and used for comparison of the discriminatory power of VNTR typing for individual loci and for all loci taken together.

## Results

Baseline clinical and sociodemographic parameters of the 187 TB patients are shown in Table 1. MIRU-ETR analysis of the 187 isolates yielded 10 clusters of indistinguishable isolates and 58 unique patterns. The codes for the 15 loci were expressed in the following order: MIRU 2, 4, 10, 16, 20, 23, 24, 26, 27, 31, 39, 40, ETR-A, ETR-B, ETR-C. Cluster sizes varied from 2 to 75 isolates. The 2 largest clusters consisted of 31 strains with the 223325173533423 profile and 75 strains with the 223325153533423 MIRU-ETR profile, respectively, both consisting of Beijing strains (Table 2). For all strains, genotyping with the 15-MIRU–ETR loci set was more informative and had a higher discriminatory power than with spoligotyping, as expected (HGDI 0.747 for MIRU vs 0.572 for spoligotyping, data not shown).

**Table 1:** Baseline clinical and sociodemographic parameters of 187 tuberculosis (TB) patients

Parameters	Isolates from civilian patients (N = 138), n (%)	Isolates from prisoners (N = 49), n (%)
Mean age, y (SD)	42.4 (14.3)	32.5 (10.6)
Sex		
Male	117 (84.8)	49 (100.0)
Female	21(15.2)	0
TB treatment		
New cases	105 (76.1)	36 (73.5)
Previously treated	33 (23.9)	13 (26.5)
HIV infected	4 (2.9)	5 (10.2)
Extensive lesions shown on radiograph	35 (25.4)	12 (24.5)
Contact with TB patient	54 (39.1)	25 (51.0)
Selected sociologic parameters		
Smoker	103 (74.6)	46 (93.9)
Alcohol consumption	121 (87.7)	39 (79.6)
Drug use	9 (6.5)	20 (40.8)

**Table 2:** Prevalence of Beijing strains in clusters (15-loci MIRU-ETR analysis)*†

Cluster no.	Size	No. repeats in MIRU-ETR loci														
		MIRU												ETR		
		2	4	10	16	20	23	24	26	27	31	39	40	A	B	C
1	3	2	2	3	1	2	5	1	4	3	3	2	4	3	2	3
2	2	2	2	3	3	2	6	1	5	3	3	2	5	3	2	3
3	3	2	2	3	3	2	5	1	5	3	4	3	3	4	2	3
4	75	2	2	3	3	2	5	1	5	3	5	3	3	4	2	3
5	3	2	3	3	3	2	5	1	5	3	5	3	3	4	2	3
6	2	2	2	1	3	2	5	1	7	3	5	3	3	4	2	3
7	31	2	2	3	3	2	5	1	7	3	5	3	3	4	2	3
8	2	2	2	3	3	2	5	1	3	3	6	3	3	4	2	3
9	2	2	2	7	2	2	5	1	1	3	2	2	3	4	2	4
10	3	1	2	4	3	2	5	1	5	3	2	2	5	2	2	1

*MIRU, mycobacterial interspersed repetitive units; ETR, exact tandem repeats. †Clusters 3–8 had 100% Beijing strains; the other clusters had none.

The results of the allelic diversity analysis for all 187 isolates are summarized in Table 3. The discriminatory index for 5 loci (MIRU 26, MIRU 31, MIRU 39, MIRU 40, and ETR-A) exceeded 0.3; these loci were regarded as moderately discriminating according to definitions proposed in a recent study (*23*). Other loci were found to be less polymorphic, with HGDIs within the range 0 to 0.3; no polymorphism was registered for loci MIRU 24 and ETR-B. The maximal number of allelic variants (*9*) was registered for locus MIRU 10, although its discriminatory power was poor because of uneven distribution of isolates with different numbers of repeats.

**Table 3:** Frequency of occurrence of MIRU-ETR alleles and allelic diversity at each locus for all strains*

Locus	No. repeats										HGDI	No. AV
	0	1	2	3	4	5	6	7	8	9		
MIRU 2	0	11	172	3	0	0	1	0	0	0	0.151	4
MIRU 4	0	2	182	3	0	0	0	0	0	0	0.053	3
MIRU 10	0	2	3	166	15	3	1	5	1	1	0.206	9
MIRU 16	0	24	7	156	0	0	0	0	0	0	0.288	3
MIRU 20	0	1	186	1	0	0	0	0	0	0	0.011	3
MIRU 23	0	2	0	0	0	173	9	3	0	0	0.142	4
MIRU 24	0	187	0	0	0	0	0	0	0	0	0	1
MIRU 26	0	7	0	2	11	122	5	40	0	0	**0.526**†	6
MIRU 27	1	0	2	184	0	0	0	0	0	0	0.032	3
MIRU 31	0	1	20	31	7	124	4	0	0	0	**0.522**	6
MIRU 39	0	0	52	135	0	0	0	0	0	0	**0.404**	2
MIRU 40	2	3	7	147	14	13	1	0	0	0	**0.372**	7
ETR-A	0	3	18	28	138	0	0	0	0	0	**0.426**	4
ETR-B	0	0	187	0	0	0	0	0	0	0	0	1
ETR-C	0	11	9	157	10	0	0	0	0	0	0.288	4

*N = 187; MIRU, mycobacterial interspersed repetitive units; ETR, exact tandem repeats; HGDI, Hunter-Gaston diversity index; AV, allelic variants. †**Boldface** loci showed at least moderate discriminative power as defined by Sola et al. (*23*) and were the most promising loci. Other loci provided poor discrimination or were monomorphic.

Spoligotyping identified 129 isolates as belonging to the Beijing family (69.0%), a proportion similar to that previously reported for Samara Oblast (*7**,**18*). Most (123) of the Beijing isolates had the characteristic spoligotyping profile with 9 final spacers present in the DR region (35–43), whereas 6 isolates had incomplete profiles because they lacked spacer 40 (4 isolates) or 43 (2 isolates). Results of spoligotyping were verified by detection of the IS*6110* insertion in the *dnaA*-*dnaN* region. All 129 isolates yielded a PCR-product ≈2,000 bp, indicative of Beijing strains. Isolates belonging to strains other then Beijing did not possess an insertion in this region and yielded an ≈550-bp PCR product.

The allelic diversity and HGDIs were calculated separately for Beijing strains (Table 4). These were subjected to VNTR analysis by using an additional panel of loci (0424, 0531, 1955, 1982, 2074, 2163a, 3232, 3239, 3336, and 3690; Table A1). The allelic diversity analysis results calculated by using 25-MIRU loci and discriminatory indices are presented in Table 5.

**Table 4:** Frequency of MIRU-ETR alleles and allelic diversity at each locus for Beijing strains only*

Locus	No. repeats										HGDI	No. AV
	0	1	2	3	4	5	6	7	8	9		
MIRU 2	0	0	127	1	0	0	1	0	0	0	0.031	3
MIRU 4	0	0	126	3	0	0	0	0	0	0	0.046	2
MIRU 10	0	2	1	126	0	0	0	0	0	0	0.046	3
MIRU 16	0	1	0	128	0	0	0	0	0	0	0.016	2
MIRU 20	0	0	129	0	0	0	0	0	0	0	0	1
MIRU 23	0	0	0	0	0	128	0	1	0	0	0.016	2
MIRU 24	0	129	0	0	0	0	0	0	0	0	0	1
MIRU 26	0	0	0	2	1	89	0	37	0	0	**0.445**†	4
MIRU 27	0	0	0	129	0	0	0	0	0	0	0	1
MIRU 31	0	1	0	1	6	117	4	0	0	0	**0.176**	5
MIRU 39	0	0	1	128	0	0	0	0	0	0	0.016	2
MIRU 40	0	0	1	127	0	1	0	0	0	0	0.031	3
ETR-A	0	0	0	3	126	0	0	0	0	0	0.046	2
ETR-B	0	0	129	0	0	0	0	0	0	0	0	1
ETR-C	0	1	0	128	0	0	0	0	0	0	0.016	2

*N = 129; MIRU, mycobacterial interspersed repetitive units; ETR, exact tandem repeats; HGDI, Hunter-Gaston diversity index; AV, allelic variants. †**Boldface** loci showed at least moderate discriminative power as defined by Sola et al. (*23*) and were the most promising loci. Other loci provided poor discrimination or were monomorphic.

**Table 5:** Frequency of an expanded set of 25 VNTR-MIRU alleles and allelic diversity for each locus for Beijing strains*†

Locus	No. repeats																			HGDI	No. AV
	1	2	3	4	5	6	7	8	9	10	12	13	14	15	16	17	20	25	26		
424	0	1	3	125	0	0	0	0	0	0	0	0	0	0	0	0	0	0	0	0.061	3
531	0	0	0	0	0	0	0	0	0	0	0	0	0	0	0	0	0	128	1	0.016	2
1955	1	4	1	122	1	0	0	0	0	0	0	0	0	0	0	0	0	0	0	0.105	4
1982	0	1	0	1	3	34	2	86	2	0	0	0	0	0	0	0	0	0	0	0.489	7
2074	0	129	0	0	0	0	0	0	0	0	0	0	0	0	0	0	0	0	0	0	1
2163a	0	1	0	0	2	5	0	1	117	1	2	0	0	0	0	0	0	0	0	0.177	7
3232	0	0	1	0	0	0	0	0	1	3	62	1	50	5	3	1	2	0	0	0.621	10
3239	0	2	126	0	1	0	0	0	0	0	0	0	0	0	0	0	0	0	0	0.046	3
3336	0	1	0	0	1	0	124	2	1	0	0	0	0	0	0	0	0	0	0	0.076	5
3690	1	128	0	0	0	0	0	0	0	0	0	0	0	0	0	0	0	0	0	0.016	2

*N = 129; VNTR, variable-number tandem repeats; MIRU, mycobacterial interspersed repetitive units; HGDI, Hunter-Gaston discriminatory index; AV, allelic variants. †No loci had allelic variants with 11, 18, 19, and 21–24 repeats.

## Discussion

This study evaluated the discriminatory ability of VNTR analysis in a population of patients with TB, in which a predominant proportion of the infecting TB strains belonged to the Beijing family. This genetic group, along with the W family, have been recently shown to share similar characteristics, including absence of spacers 1–34 in the DR region of the *M. tuberculosis* genome (*14**,**15**,**17*). The latter characteristic, although well known and widely accepted, may not always be definitive for identification of Beijing/W strains alone, as spacers 35–43 are present in several other *M. tuberculosis* families. Moreover, some spacers (particularly 37, 38, and 40) are missing in certain Beijing/W isolates because of loss of the target for DRa and DRb primers caused by deletions or presence of IS*6110* insertions in the DR region (*32**,**33*).

Results of spoligotyping were confirmed by detection of an IS*6110* insertion between the *dnaA* and *dnaN* genes, which was found in all 129 Beijing isolates but not in the remaining isolates. Results of our analysis demonstrate 100% specificity of the method and its applicability for robust, rapid, and reliable identification of Beijing family strains.

In our study, 6 (4.7%) of 129 isolates identified as Beijing on the basis of spoligotyping had incomplete spoligotyping profiles with a missing spacer 40 or 43. As has been argued by Bifani et al. (*32*), W strains with missing spacer 40 are defined as W14 group, characterized by a specific IS*6110* RFLP pattern, high levels of drug resistance, and an additional repeat in ETR-D (MIRU 4) locus, i.e., MIRU-ETR profile 23332515 (or 7) 3533423. However, we observed no differences in the number of repeats in the MIRU 4 locus for these 4 isolates. All 4 isolates were multidrug resistant strains. Of the 4 patients, all were male, 1 was HIV-infected, 2 were Russian (1 from Chechnya and 1 from Azerbaijan), and 2 had been vaccinated with BCG.

The data from the application of 15-MIRU–ETRs demonstrated the homogeneity and clonality of TB strains in this region of Russia: 135 (72.2%) of 187 strains were clustered into 10 groups, which indicates high rates of recent TB transmission in the Samara region. General trends for VNTR loci diversity and discriminatory power agreed (with some exceptions) with those reported previously (*20**,**22**,**23**,**34*). The overall allelic polymorphism and discriminatory power of the VNTR loci in this population were lower than that reported in previously published studies because of the large number of Beijing isolates in our test panel. This number reflects the actual prevalence of Beijing strains in Samara Oblast and in the few other regions in Russia for which the proportion of Beijing strains has been described (Table 2, Table 3, Table 4).

The 15-MIRU–ETR profiles 223325153533423 or 223325173533423 were shared by 106 (82.2%) of the total number of analyzed Beijing strains with an HGDI of 0.625 for all 15 loci. Four loci (MIRU20, MIRU24, MIRU27, and ETR-B) were monomorphic for Beijing strains, and the only locus with sufficient discriminatory power for differentiating among Beijing family strains was MIRU 26. This finding is in marked contrast to the application of MIRU-ETR in other patient populations in which the discriminatory power of MIRU, used in conjunction with spoligotyping, was arguably almost comparable to RFLP IS*6110* (*20**–**23**,**35*). In these studies, HGDI values were subdivided into 3 groups, according to their ability to discriminate: poor (2, 20, 27), moderate (4, 16, 24, 39, ETR-B, ETR-C), and high (10, 23, 26, 31, 40, ETR-A) (*23*).

We expanded the panel of loci to improve discrimination among the Beijing isolates by using loci that had previously been reported to be highly polymorphic (*24**–**27*). The allelic diversity analysis and discriminatory indices are presented in Table 5.

The expanded set of VNTRs provided better discrimination than the original set of 15-MIRU–ETRs: 53 different profiles were identified, including 9 shared types in clusters (2–46 isolates each) and 44 unique patterns. The HGDI for 25 VNTR loci together was 0.870 (compared with 0.625 for the original set of MIRU-ETRs).

The most discriminatory individual VNTR were loci 3232 and 1982, with a large number of allelic variants (10 and 7, respectively) and HGDIs of 0.621 and 0.489, respectively. Other loci demonstrated poor discriminatory power (HGDI 0–0.2), and locus 2074 was monomorphic.

In our analysis of Russian Beijing isolates, 3 loci (MIRU 26, VNTR 1982, and VNTR 3232; 0.3–0.6) were sufficiently polymorphic for differentiation within the Beijing family. A relatively high degree of polymorphism in MIRU 26 has been previously reported (*20**,**23**,**36*). Loci 3232 and 1982 have been much less studied. Locus 3232 was shown to be more polymorphic in *M. tuberculosis* than in *M. bovis* (8 vs. 5 allelic variants, respectively) with moderate to high HGDI values (0.60). Locus 3232 displayed the highest discrimination power among 8 novel VNTR loci introduced by Roring et al. (*24**,**25*). The basis of polymorphism in loci MIRU 26, VNTR 3232, and VNTR 1982 is not completely clear. The first 2 loci are located in the intergenic regions of genes involved in metabolism of cell membrane components and transmembrane transport (locus VNTR 3232) and between genes *Rv2679* (possible *echA15*, enoyl-CoA hydratase) and *Rv 2680* (unknown protein) (locus MIRU 26). Locus VNTR 1982 is part of the *Rv1753c* (PPE24), which belongs to the group of genes encoding PPE proteins and which has been argued to be responsible for antigenic variation of *M. tuberculosis* (*37*). In our study, the degree of polymorphism at this locus was considerably higher than that previously reported for a panel of *M. bovis* isolates (*27*). By contrast, loci 2163a, 1955, 3336, and 3690, which were reported to be polymorphic for *M. bovis*, demonstrated low polymorphism in our study of *M. tuberculosis* Beijing isolates.

We speculate that the presence of variation at a small number of loci in genes likely to be involved in the earliest interactions of pathogen and host against a background of homogeneity at other loci suggests that these regions may be involved in the successful transmission of Beijing family strains. For the whole population studied, a small group of loci (VNTR 1982, VNTR 3232, MIRU 10, MIRU 26, MIRU 31, MIRU 39, MIRU 40, and ETR-A) offered the most discriminating panel and may be considered an essential part of the prospective universal panel of VNTR loci suitable for differentiation of *M. tuberculosis* isolates in different geographic settings with variable prevalence of highly conserved genotypes. The application of a limited number of loci (MIRU 10, MIRU 26, MIRU 31, VNTR 3232, and VNTR 1982) independently or in combination with other loci, may be a useful and rapid tool for differentiating strains within the Beijing family and for practical prospective genotyping and tracing of TB outbreaks in populations where the Beijing genotype predominates. Given the limited number of loci, analyzing this panel with a manual or automated approach is practical.

In conclusion, our study shows that by expanding the VNTR panel beyond the 15-MIRU–ETR loci described previously, discrimination can be substantially increased. This method may be used for typing *M. tuberculosis* isolates, even for populations in which a particular genetic group is dominant.

## Appendix

**Table A1:** Extended panel of loci, primer sequences, and PCR conditions for VNTR-MIRU analysis*

	Markers					Primer sequences		
	H37Rv locus name	MIRU system† (*1**,**2*)	ETR system (*3*)	Mtub system (*4*)	QUB system (*5**,**6*)	Forward	Reverse	Label
1	0154	MIRU2†				5´-CAG GTG CCC TAT CTG CTG ACG-3´	5´-GTT GCG TCC GGC ATA CCA AC-3´	Dye 3
2	0424			Mtub4		5´-CCG CCC TGG TCG TCT GGA-3´	5´-CGG CAT CCT CAA CAA CGG TAG C-3´	Dye 3
3	0531		MPTR-A			5´-CTG CCG AGG CCC GCG TTG ATT GT-3´	5´-GAC GTG GCG GCG ATG GCT GGT TA-3´	Dye 2
4	0577		ETR-C‡			5´-GTG AGT CGC TGC AGA ACC TGC AG-3´	5´-GGC GTC TTG ACC TCC ACG AGT G-3´	Dye 3
4	0580	MIRU4	EDR-D			5´-GTC AAA CAG GTC ACA ACG AGA GGA A-3´	5´-CCT CCA CAA TCA ACA CAC TGG TCA T-3´	Dye 3
5	0802	MIRU40				5´-GAT TCC AAC AAG ACG CAG ATC AAG A-3´	5´-TCA GGT CTT TCT CTC ACG CTC TCG-3´	Dye 3
6	0959	MIRU10				5´-ACC GTC TTA TCG GAC TGC ACT ATC AA-3´	5´-CAC CTT GGT GAT CAG CTA CCT CGA T-3´	Dye 4
7	1644	MIRU16				5´-CGG GTC CAG TCC AAC TAC CTC AAT-3´	5´-GAT CCT CCT GAT TGC CCT GAC CTA-3´	Dye 2
8	1955			Mtub21		5´-TGT CGA GTT CAC CGT CCA TCA TCT-3´	5´-CCG ACG CCA ATA GCA CAG CAC CAG-3´	Dye 4
9	1982				QUB-18	5´-GGA ATG GCT ACG GAA GGA ATA CTC-3´	5´-TTA CGA CAC CTG ATC TGA CTC TGC- 3´	Dye 2
10	2059	MIRU20				5´-CCC CTT CGA GTT AGT ATC GTC GGT T-3´	5´-CAA TCA CCG TTA CAT CGA CGT CAT C-3´	Dye 2
11	2074			Mtub24		5´-CGC GAG GAC GAG GTG GAG AA-3´	5´-ACA ATT GCA GCC AGA GAT GAG ACG-3´	Dye 4
12	2163a				QUB-11a	5´-CCC GGG GCG CTC GTG ATG- 3´	5´-CGG CGG CAC CCT GGA GTC TGG-3´	Dye 4
13	2165		ETR-A‡			5´-AAA TCG GTC CCA TCA CCT TCT TAT-3´	5´-CGA AGC CTG GGG TGC CCG CGA TTT-3´	Dye 2
14	2387	MIRU24				5´-GAA GGC TAT CCG TCG ATC GGT T-3´	5´-GGG CGA GTT GAG CTC ACA GAA C-3´	Dye 3
15	2461		ETR-B‡			5´-GCG AAC ACC AGG ACA GCA TCA TG-3´	5´-GGC ATG CCG GTG ATC GAG TGG-3´	Dye 4
16	2531	MIRU23				5´-CGA ATT CTT CGG TGG TCT CGA GT-3´	5´-ACC GTC TGA CTC ATG GTG TCC AA-3´	Dye 4
17	2996	MIRU26				5´-GCG GAT AGG TCT ACC GTC GAA ATC-3´	5´-TCC GGG TCA TAC AGC ATG ATC A-3´	Dye 4
18	3006	MIRU27			QUB-5	5´-TCT GCT TGC CAG TAA GAG CCA-3´	5´-GTG ATG GTG ACT TCG GTG CCT T-3´	Dye 4
19	3192	MIRU31	ETR-E			5´-CGT CGA AGA GAG CCT CAT CAA TCA T-3´	5´-AAC CTG CTG ACC GAT GGC AAT ATC-3´	Dye 3
20	3232				QUB-3232	5´-CAC TAG TTG TTG CGG CGA TGG T-3´	5´-AAG GGC GGC ATT GTG TTC C-3´	Dye 3
21	3239		ETR-F			5´-GAC TTC GGG CAG CTC GGG CAT CC-3´	5´-CCG CGG TGG TTG TCG TGA TG-3´	Dye 2
22	3336				QUB-3336	5´-GAT CGG GTG CAG TGG TTT CAG GTG-3´	5´-GGG CGG CCA GCG GTG TC-3´	Dye 3
23	3690			Mtub39		5´-CGA GGA TCA CGA TGC GGG TCA C-3´	5´-GGC GGG GGC TCG GGT GGT A-3´	Dye 4
24	4348	MIRU39				5´-CGG TCA AGT TCA GCA CCT TCT ACA TC-3´	5´-GCG TCC GTA CTT CCG GTT CAG-3´	Dye 2

*VNTR, variable number tandem repeats; MIRU, mycobacterium interspersed repetitive units, ETR, exact tandem repeats. †Primer sequences by Kwara et al. (*7*). ‡Primer sequences by Frothingham et al. (*3*).
